# *Csn5* Is Required for the Conidiogenesis and Pathogenesis of the *Alternaria alternata* Tangerine Pathotype

**DOI:** 10.3389/fmicb.2018.00508

**Published:** 2018-03-20

**Authors:** Mingshuang Wang, Xiao Yang, Ruoxin Ruan, Huilan Fu, Hongye Li

**Affiliations:** ^1^Key Lab of Molecular Biology of Crop Pathogens and Insects, Institute of Biotechnology, Zhejiang University, Hangzhou, China; ^2^Hangzhou Academy of Agricultural Sciences, Hangzhou, China

**Keywords:** *csn5*, conidiation, pathogenesis, *Alternaria alternata*, tangerine pathotype, RNA-Seq

## Abstract

The COP9 signalosome (CSN) is a highly conserved protein complex involved in the ubiquitin-proteasome system. Its metalloisopeptidase activity resides in subunit 5 (CSN5). Functions of *csn5* in phytopathogenic fungi are poorly understood. Here, we knocked out the *csn5* ortholog (*Aacsn5*) in the tangerine pathotype of *Alternaria alternata*. The Δ*Aacsn5* mutant showed a moderately reduced growth rate compared to the wildtype strain and was unable to produce conidia. The growth of Δ*Aacsn5* mutant was not affected in response to oxidative and osmotic stresses. Virulence assays revealed that Δ*Aacsn5* induced no or significantly reduced necrotic lesions on detached citrus leaves. The defects in hyphal growth, conidial sporulation, and pathogenicity of Δ*Aacsn5* were restored by genetic complementation of the mutant with wildtype *Aacsn5*. To explore the molecular mechanisms of conidiation and pathogenesis underlying *Aacsn5* regulation, we systematically examined the transcriptomes of both Δ*Aacsn5* and the wildtype. Generally, 881 genes were overexpressed and 777 were underexpressed in the Δ*Aacsn5* mutant during conidiation while 694 overexpressed and 993 underexpressed during infection. During asexual development, genes related to the transport processes and nitrogen metabolism were significantly downregulated; the expression of *csn1–4* and *csn7* in Δ*Aacsn5* was significantly elevated; secondary metabolism gene clusters were broadly affected; especially, the transcript level of the whole of cluster 28 and 30 was strongly induced. During infection, the expression of the host-specific ACT toxin gene cluster which controls the biosynthesis of the citrus specific toxin was significantly repressed; many other SM clusters with unknown products were also regulated; 86 out of 373 carbohydrate-active enzymes responsible for breaking down the plant dead tissues showed uniquely decreased expression. Taken together, our results expand our understanding of the roles of *csn5* on conidiation and pathogenicity in plant pathogenic fungi and provide a foundation for future investigations.

## Introduction

The COP9 signalosome (CSN) is an evolutionarily conserved protein complex initially identified as a photomorphogenic regulator of development in *Arabidopsis thaliana* (Wei et al., 1994) and subsequently characterized in mammals, invertebrates, and fungi (Oron et al., 2002; Kato and Yoneda-Kato, 2009; Braus et al., 2010). The CSN complex regulates ubiquitin-proteasome-mediated proteolysis. The best-studied function of the CSN is the control of the activity of cullin–RING ubiquitin E3 ligases (CRLs; Petroski and Deshaies, 2005). A typical CRL complex consists of a backbone cullin subunit, a RING protein, an adaptor protein, and a substrate-recognition subunit (Petroski and Deshaies, 2005). Nedd8 (neural precursor cell expressed, developmentally downregulated 8) is a ubiquitin-like protein which promotes the assembly of the CRL complex and the ubiquitylation of substrates by covalently attaching to a conserved lysine site on cullin proteins. The CSN functions as an isopeptidase enzyme to negatively regulate the activity of CRLs by removing the Ndedd8 from cullins (deneddylation) (Lyapina et al., 2001).

The CSN complex is composed of eight subunits in higher eukaryotic organisms, designated as CSN1–CSN8 (Wei and Deng, 2003). In humans, the CSN has two organizational centers: a horseshoe-shaped ring formed by CSN1–CSN4 and CSN7–CSN8 which contain a PCI (proteasome lid-CSN-initiation factor 3) domain and a large carboxy-terminal helical bundle created by all the subunits. CSN5 and CSN6, which have an MPN (Mpr1 and Pad1 N-terminal) domain, form a dimer and are embedded at the core of the helical bundle (Lingaraju et al., 2014). Specifically, the CSN5 subunit harbors a metalloprotease motif (JAMM, Jab1/MPN/Mov34) in the MPN domain responsible for the isopeptidase activity, making it the sole subunit that can provide the catalytic center for the CSN complex and cleave the Ndedd8 from cullins (Cope et al., 2002). Besides the basic role in regulating the activity of CRLs, the CSN complex also participates in many other biological processes. The botanic CSN is involved in photomorphogenesis, hormone signaling, flower development, iron deficiency, and plant pathogen response (Craig et al., 2009; Santner and Estelle, 2010; Lau and Deng, 2012; Stratmann and Gusmaroli, 2012; Duplan and Rivas, 2014; Tan et al., 2016). In mammals, the CSN is involved in several important biological functions such as cell cycle regulation, checkpoint control, signal transduction, autophagy, apoptosis, and tumorigenesis (Kato and Yoneda-Kato, 2009; Lee et al., 2011; Su et al., 2013).

The subunit composition of CSN in some fungi can be significantly different from that of the higher organisms. *Neurospora crassa* lacks the CSN8 subunit while the fission yeast *Schizosaccharomyces pombe* lacks CSN6 and CSN8 (Mundt et al., 2002; Liu et al., 2003; Wang et al., 2010). The budding yeast *Saccharomyces cerevisiae* possesses an alternative CSN complex composed of six subunits (Maytal-Kivity et al., 2002). In higher eukaryotes, the absence of any subunit is lethal in early stages of development (Wei et al., 2008; Zhao et al., 2011). However, fungi can survive without a CSN subunit (Gerke and Braus, 2014). Therefore, fungi are ideal model systems for understanding the evolution and biological roles of CSN. Functions of the fungal CSN have been characterized in several model species. In *S. pombe*, the CSN is required for the coordination of S phase and resistance to DNA damage (Mundt et al., 1999, 2002). In *S. cerevisiae*, the CSN plays a role in mating efficiency, pheromone sensitivity, lipid metabolism, and zinc absorption (Maytal-Kivity et al., 2002; Licursi et al., 2014). The *N. crassa* CSN is essential for conidiation and circadian rhythms (Wang et al., 2010), while the CSN in *Aspergillus nidulans* is involved in the light response, sexual development, and secondary metabolism (SM; Nahlik et al., 2010). Despite these advances, the molecular mechanism underlying the CSN’s control of development, pathogenicity, and SM in filamentous phytopathogenic fungi is poorly understood.

*Alternaria alternata* is a necrotrophic fungus, which can be ubiquitously isolated from soil and various plants and decaying plant materials (Thomma, 2003). Some of the strains in this species can cause diseases on plants and result in severe crop losses worldwide. At least seven pathogenic *A. alternata* pathotypes, each producing a unique host-selective toxin (HST), have been recognized to cause diseases in Japanese pear, strawberry, tangerine, apple, tomato, rough lemon, and tobacco, respectively (Tsuge et al., 2013). The HSTs in *A. alternata* pathotypes are crucial for their respective pathogenicity (Tsuge et al., 2013). The tangerine pathotype of *Alternaria alternata* is the causal agent of the Alternaria brown spot disease, which causes significant losses of both yield and marketability for tangerines and tangerine hybrids worldwide. In this study, we have identified the *csn5* gene homolog in the tangerine pathotype of *A. alternata* (*Aacsn5*) and characterized its function using a gene deletion and complementation strategy. We also performed a transcriptome-wide gene expression analysis to explore the regulatory role of *Aacsn5* in the development of conidia and pathogenicity in this important citrus pathogen.

## Materials and Methods

### Fungal Strains and Culture Conditions

The reference *A. alternata* strain, Z7, was isolated from an infected citrus fruit from Zhejiang, China (Huang et al., 2015; Wang et al., 2016). Z7 and its derived mutants were stored in 20% glycerol solutions at -80°C until use. Fungi were grown on regular solid PDA (potato dextrose agar) at 25°C and conidia were collected after incubating for 8 days. Mycelia were obtained by growing spores in liquid potato dextrose broth (PDB) incubated on a rotary shaker at 160 rpm at 25°C for 2 days.

### Genetic Construction of *Aacsn5* Deletion and Complementation Mutants

The *Aacsn5* (accession number AALT_g2342) was identified using the *A. nidulans* (accession number AAM95164) *csn5* homolog as a query to search the proteome of *A. alternata* by BLASTp. The *Aacsn5* gene was knocked out using a fungal protoplast transformation as described previously (Chen et al., 2017; Ruan et al., 2017). Briefly, the deletion vectors were constructed by inserting two ∼1 kb flanking sequences of the *Aacsn5* gene into the left and right sides of the neomycin-resistance gene in the pA1300-NEO plasmid (Wang et al., 2015). The resulting fragment was then amplified and directly introduced into the wildtype protoplasts using polyethylene glycol and CaCl_2_ (Supplementary Figure S2A). Transformants growing on a regeneration medium amended with 100 μg/mL neomycin were selected, examined by polymerase chain reaction (PCR) with specific primer pairs, and verified by southern blot (Supplementary Figures S2B,C). For genetic complementation, a full-length *Aacsn5* fragment with its endogenous promoter was amplified by PCR and was inserted into the pTFCM plasmid carrying a hygromycin-resistance gene (Wang et al., 2015). The resultant plasmid was then transformed into protoplasts of a Δ*Aacsn5* mutant. Transformants were recovered from medium supplemented with hygromycin (150 mg/L) and examined by PCR and southern blot (Supplementary Figures S2B,C). All the primers used in this study are listed in Supplementary Table S1.

### Fungal Growth Assays and Ergosterol Quantification

To examine oxidative and osmotic stress tolerance, Δ*Aacsn5*, CP*Aacsn5*, and wildtype *A. alternata* were grown on PDA plates supplemented with either 1.5 M NaCl, 0.5 M CaCl_2_, 1 mM H_2_O_2_, or 3 mM menadione (VK_3_). Each plate was inoculated with a 5 mm mycelial plug taken from the edge of a 5-day-old colony. The diameters of the colonies were measured after the plates were incubated at 25°C for 5 days. To examine differences in carbon and nitrogen utilization, fungal strains were grown on modified Czapek’s medium (30 g glucose, 3.0 g NaNO_3_, 1.0 g KH_2_PO_4_, 0.5 g MgSO_4_, 0.5 g KCl, 0.01 g FeSO_4_, and 15 g agar per liter). More specifically, the sole source of carbon (i.e., glucose) was replaced with equal mass sucrose, cellulose, hemicellulose, pectin, or citrus leaf to examine differences in carbon utilization and the sole source of nitrogen (i.e., NaNO_3_) was replaced with equal mass yeast extract, peptone, urea, acetamide, or phenylalanine to examine differences in nitrogen utilization. The diameters of the colonies were measured after the plates were incubated at 25°C for 6 days. For ergosterol extraction, fungal strains were added to a 150 mL yeast glucose (YG) medium (5 g/L yeast extract and 15 g/L glucose) and incubated at 25°C for 2 days on a rotary shaker. Mycelia were harvested by passing through a filter paper and washing three times with sterile water. Total ergosterol was extracted with hexane using a previously described method (Wang et al., 2014). Ergosterol samples were analyzed using an Agilent1100 high-performance liquid chromatography system. All experiments were repeated two times.

### Pathogenicity and Toxin Assays

Fungal virulence was assessed by placing a 5 mm plug taken from the PDA media on ponkan (*Citrus reticulata Blanco*) leaves for 48 h or by placing the mycelia harvested from the liquid PDB media on the leaves for 24 h. Each strain was tested on at least 15 leaves and experiments were repeated two times. Host-selective ACT toxin was crudely extracted using Amberlite XAD-2 resin and ethyl acetate from culture filtrates (Kohmoto et al., 1993). The toxicity was assessed by spreading 40 μl ethyl acetate extracts onto detached ponkan leaves.

### Transcriptome Analysis

To investigate the regulatory role of *Aacsn5* in conidia formation, the colonies of wildtype and Δ*Aacsn5* mutants were scraped from the solid PDA medium after the incubation for 7 days at which time the conidia of the wildtype began to form. To investigate the regulatory role of *Aacsn5* in pathogenesis, an equal number of mycelia from both wildtype and Δ*Aacsn5* mutants were inoculated on the citrus leaves, respectively, and were harvested at 24 h post-inoculation. Collected mycelia of each sample were then ground in liquid nitrogen and total RNA was extracted using an AxyPrepTM multisource total RNA miniprep kit. RNA-Seq was conducted for two biological replicates of each sample. The libraries were performed using an IlluminaTruSeq RNA Sample Preparation Kit and were sequenced on an Illumina Hiseq 2500 platform, generating 150 bp paired-end reads. Raw RNA-Seq reads were trimmed of adapter sequences and low-quality reads using trimmomatic (Bolger et al., 2014). Index of the *A. alternata* Z7 genome was built using Bowtie2 (Langmead and Salzberg, 2012) and cleaned reads were mapped to the reference genome using TopHat2 (Kim et al., 2013). The number of reads mapped to each gene was counted by HTSeq (Anders et al., 2014) and the resulting transcript count tables were subjected to DESeq R package for differential expression analysis (Anders and Huber, 2012). Transcripts with an adjusted *P* value less than 0.01 and the absolute value of log2FC (log2 fold change) greater than 2 were determined as differentially expressed. The differentially expressed genes (DEGs) were annotated by BLAST search against the NCBI non-redundant protein sequences database. Gene ontology (GO) enrichment analysis of DEGs was conducted using topGO (Alexa and Rahnenfuhrer, 2016). Kyoto Encyclopedia of Genes and Genomes (KEGG) pathway analysis was performed using KOBAS 3.0 (Xie et al., 2011). The transcriptome data reported in this study have been deposited in NCBI’s Sequence Read Archive with accession number SRP120511.

### qRT-PCR Analysis

To validate the transcriptome data obtained by RNA sequencing, quantitative real-time PCR (qRT-PCR) was carried out on 12 *A. alternata* genes; 5 μg of each RNA sample was used for reverse transcription with the Prime Script RT reagent kit (TakaRa Biotechnology, Co., Dalian, China). The relative transcript level of the selected genes was quantified in triplicate on a 7300 Real-Time PCR system (ABI, United States). Primers used in this study were listed in Supplementary Table S1. The actin-encoding gene (KP341672) was used as an internal control and the resulting data were normalized using the comparative 2^-ΔΔC_T_^ as described previously (Wang et al., 2014).

## Results

### Identification and Deletion of *Aacsn5*

By searching the whole genome of *A. alternata* Z7, we discovered a gene showing 58% amino acid identity and 97% coverage to the *A. nidulans* CSN5 homolog, and thus we designated this gene *Aacsn5*. Sequence alignment of CSN5 homologs from fungal species with different taxonomies confirmed that they share a highly conserved MPN domain (Supplementary Figure S1). The cloned *Aacsn5* gene, which encodes a protein of 353 amino acids, has a 1149 bp ORF interrupted by one intron of 81 bp.

To investigate the functions of *Aacsn5*, an *A. alternata* mutant defective for *Aacsn5* was generated by targeted gene disruption (Supplementary Figure S2A). In the mutant *Aacsn5* allele, a 637 bp fragment spanning from Ala-55 to Arg-267 including the conserved MPN domain was replaced by the neomycin-resistance cassette. The disrupted and complemented mutants were first verified by PCR analysis with outside and inside primer pairs (Supplementary Figure S2B) and then further confirmed by southern blot analysis (Supplementary Figure S2C). As both mutants we got showed identical phenotype, only one mutant stain was used to perform the following experiments.

### Involvement of *Aacsn5* in Vegetative Growth and Conidiation

**FIGURE 1:**
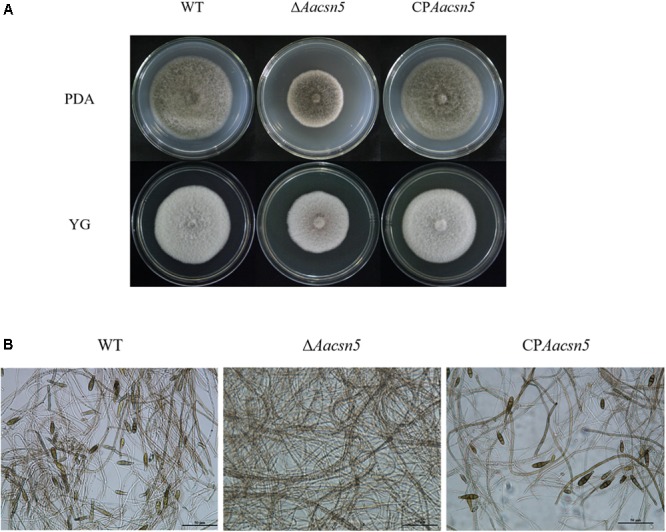
*Aacsn5* is required for vegetative growth and conidiation. **(A)** Vegetative growth of the wildtype, Δ*Aacsn5*, and CP*Aacsn5* mutant strains on PDA or YG at 25°C for 5 days. **(B)** Light microscopy images of the formation of conidia by *A. alternata* strains on PDA.

During growth on basic PDA medium, the Δ*Aacsn5* exhibited a moderate growth defect (29–34% reduction in growth) compared to the wildtype strain (**Figure 1A**). A similar phenotype (18–23% reduction in growth) was also observed when the Δ*Aacsn5* mutant was grown on the YG (yeast 5 g, glucose 15 g, 1 L) medium (**Figure 1A**). The defects in hyphal growth of Δ*Aacsn5* on solid media were restored by genetic complementation of the mutant with wildtype *Aacsn5* (**Figure 1A**). Conidiation is very important for ascomycetous fungi to survive adversity and propagate. The wildtype strain Z7 can produce a large number of conidia after incubation in solid PDA for 7 days; however, no conidia were observed in the Δ*Aacsn5* mutant (**Figure 1B**). The Δ*Aacsn5* mutant was allowed to grow for two additional weeks but did not produce any conidia during this time period, demonstrating that conidiation is entirely abrogated and not merely delayed in this mutant. Thus, *Aacsn5* is absolutely required for the formation of conidia in *A. alaternata*. The defects in conidial sporulation of Δ*Aacsn5* on solid media were partly (∼50%) restored by genetic complementation of the mutant with wildtype *Aacsn5* (**Figure 1B**).

### *Aacsn5* Is Dispensable for Stress Response and Sterol Biosynthesis

**FIGURE 2:**
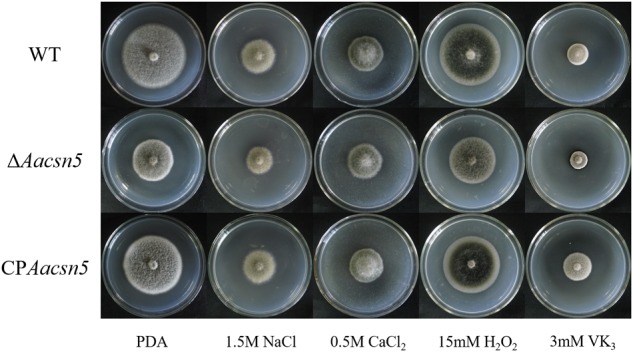
Effect of osmotic and oxidative stresses on the growth of the wildtype, Δ*Aacsn5*, and CP*Aacsn5* mutant strains. Mycelial plugs were taken from the wildtype, Δ*Aacsn5*, and CP*Aacsn5* mutants and grown individually on PDA medium supplemented with NaCl, CaCl_2_, H_2_O_2_, and menadione at the concentrations indicated in the figure. All plates were incubated for 5 days at 25°C.

To investigate whether *Aacsn5* is involved in external stress tolerance, wildtype and mutant strains were grown on PDA media supplemented with salt stress inducers (1.5 M NaCl and 0.5 M CaCl_2_) and with oxidative stress inducers (15 mM H_2_O_2_ and 3 mM VK_3_) and analyzed for growth defects. No obvious change in stress tolerance was detected between the Δ*Aacsn5* strain and wildtype (**Figure 2**), indicating that *Aacsn5* is not involved in the *A. alternata* response to osmotic and oxidative stresses.

Previously, it was reported that the *csn5* homolog in *S. cerevisiae* is involved in the regulation of ergosterol metabolism (Licursi et al., 2014). To determine whether *Aacsn5* regulates ergosterol, we quantified the total ergosterol content of the *A. alternata* wildtype and the Δ*Aacsn5* deletion mutant strains. However, no significant differences were found between them (2.25 μg/mg in the mutant versus 2.34 μg/mg in the wildtype).

### *Aacsn5* in Required for Full Pathogenicity of *A. alternata*

**FIGURE 3:**
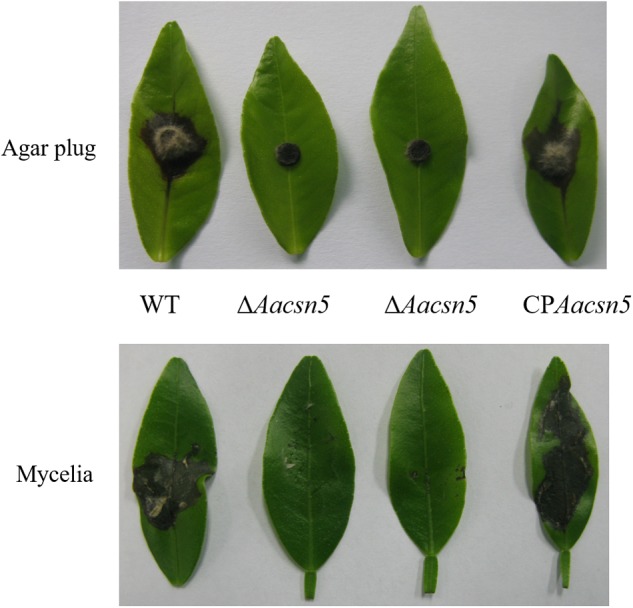
The *Aacsn5* gene is essential for fungal pathogenicity in the citrus. Necrosis symptoms in ponkan leaves inoculated with the PDA agar plug (48 h) and mycelia harvested from the liquid PDB (24 h) of the indicated strains.

To study the possible effect of *Aacsn5* on *A. alternata* virulence, infection assays were carried out on ponkan leaves. Most (26/30) ponkan leaves did not form necrotic lesions after inoculation with a Δ*Aacsn5* agar plug compared to all leaves inoculated with the wildtype strain, while four leaves formed significantly smaller lesions compared to the wildtype (**Figure 3**). To further test the virulence of the Δ*Aacsn5* mutant, we inoculated ponkan leaves with Δ*Aacsn5* and wildtype mycelia harvested from liquid PDB. We found that necrotic lesions caused by the wildtype mycelia were formed much faster than those caused by the agar plug and all leaves inoculated with Δ*Aacsn5* mycelia formed necrotic lesions (**Figure 3**). However, the Δ*Aacsn5* lesions were much less severe than those induced by the wildtype strain (**Figure 3**). Wounding the leaves prior to inoculation did not facilitate infection and lesion formation by the mutants (Supplementary Figure S3), suggesting that *Aacsn5* is not involved in the capability of the fungus to penetrate the host. Taken together, our results indicate that *Aacsn5* is required for the full virulence of *A. alternata* tangerine pathotype.

### *Aacsn5* Regulates Expression of Genes During Conidiation and Plant–Pathogen Interaction

**FIGURE 4:**
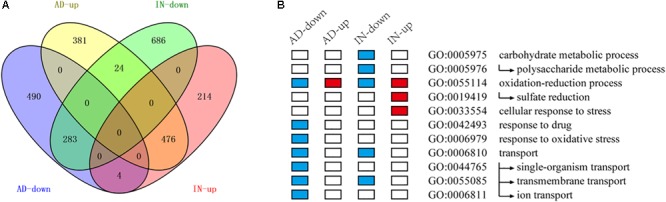
The RNA-seq analysis of Δ*Aacsn5* mutant during asexual development (AD) and infection (IN). **(A)** Venn diagram showing the upregulated and downregulated genes in Δ*Aacsn5* mutant compared with the wildtype strain. **(B)** Enriched functional categories for genes differentially expressed under different conditions. Red and blue boxes indicated GO terms enriched in overexpressed genes and underexpressed genes, respectively.

To explore the molecular mechanisms underlying the *Aacsn5* regulation of conidiation, we performed a transcriptomic analysis comparing gene expression of the wildtype and Δ*Aacsn5* strains during asexual development. Overall, 1658 genes were differentially expressed in Δ*Aacsn5* compared to the wildtype strain (Supplementary Table S2), comprising 881 overexpressed and 777 underexpressed genes (**Figure 4A**). Genes expressed at a lower degree were enriched for the functional categories TRANSPORT, RESPONSE TO OXIDATIVE STRESS, RESPONSE TO DRUG, and OXIDATION–REDUCTION PROCESS while those expressed at a higher degree were enriched in the category OXIDATION–REDUCTION PROCESS (**Figure 4B** and Supplementary Table S3). After assigning these DEGs to the KEGG pathways, under-represented genes were significantly enriched in the nitrogen metabolism pathway while overexpressed genes were significantly enriched in the riboflavin metabolism, tyrosine metabolism, sulfur metabolism, and non-homologous end-joining pathways (Supplementary Table S3).

To further explore the effect of *Aacsn5* on gene regulation during infection, we compared the transcriptomes of the Δ*Aacsn5* with the wildtype inoculated on ponkan leaves for 24 h. A total of 1687 DEGs were identified (Supplementary Table S2), comprising 694 overexpressed and 993 underexpressed genes (**Figure 4A**). Genes expressed at a lower degree were enriched for the functional categories CARBOHYDRATE METABOLIC PROCESS, TRANSPORT, and OXIDATION–REDUCTION PROCESS while those expressed at a higher degree were enriched in the category CELLULAR RESPONSE TO STRESS (**Figure 4B** and Supplementary Table S3). After assigning these DEGs to the KEGG pathways, underexpressed genes were significantly enriched in starch and sucrose metabolism, pentose and glucuronate interconversions, galactose metabolism, tyrosine metabolism, cyanoamino acid metabolism, phenylalanine metabolism, glycerolipid metabolism, fructose and mannose metabolism, arginine and proline metabolism, other glycan degradation, glycosphingolipid biosynthesis, and ascorbate and aldarate metabolism pathways while overexpressed genes were significantly enriched in the sulfur metabolism and non-homologous end-joining pathways (Supplementary Table S3).

After comparing two sets of DEGs related to conidiation and pathogenicity, 283 and 476 DGEs were upregulated and downregulated in both conditions, respectively, while 28 DEGs were oppositely regulated in both conditions (**Figure 4A**). More overexpressed genes overlapped between the two conditions (corresponding to 54% of all overexpressed genes during conidiation and 69% of all overexpressed genes during infection) than underexpressed genes (36% of all underexpressed genes during both conidiation and infection; **Figure 4A**).

To validate the RNA-seq results, 12 genes were selected and their expression levels were analyzed by qRT-PCR using gene-specific primers. These results showed that though the magnitude of fold changes between the two methods for some of the genes in the two conditions varied, overall both showed similar trends in transcript accumulation (Supplementary Figure S4).

### *Aacsn5* Affects the Expression of the CSN Complex During Asexual Development

**FIGURE 5:**
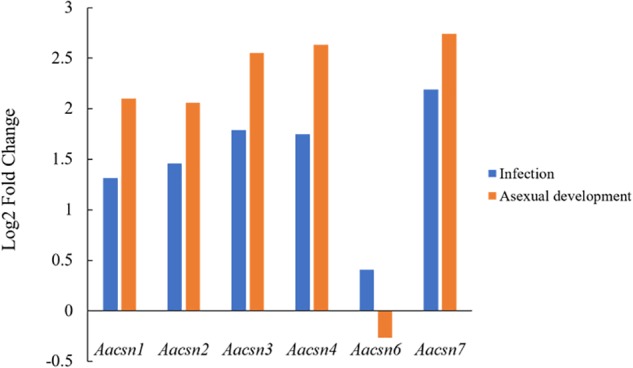
Expression of other *csn* subunit genes in the Δ*Aacsn5* mutant compared with wildtype strain during AD and IN.

The CSN complex is composed of at most eight subunits in fungi. To understand the expression pattern of these subunits in the absence of *Aacsn5*, we first searched the proteome of *A. alternata* Z7 by BLASTp using the *A. nidulans* CSN subunit proteins as queries. Like *N. crassa*, the *A. alternata* genome does not encode a *csn8*-like gene (Supplementary Table S4). As presented in **Figure 5**, with the exception of *Aacsn6*, the expression of other *csn* subunit genes in the Δ*Aacsn5* mutant was increased during infection and even more elevated during conidiation, indicating a negative feedback effect of *Aacsn5* on the expression of *csn1–4* and *csn7*, which may be involved in the conidiation process.

### *Aacsn5* Does Not Mediate the Known Mechanism Underlying Sporulation in Other Fungi

In some model fungal species like *A. nidulans* and *N. crassa*, the BrlA > AbaA > WetA central regulatory pathway, the light-dependent signaling pathway, velvet family proteins, MAP kinase signaling pathway, G proteins, and FluG-mediated signaling pathway are important regulators of asexual development (Park and Yu, 2012). After looking into the expression pattern of these genes in the wildtype and Δ*Aacsn5* mutant, however, no significant difference was found between them except for the homolog of the *flbC* gene (AALT_g5513), which was expressed at an approximately eightfold lower level during asexual development in the Δ*Aacsn5* compared to wildtype (Supplementary Table S5).

### *Aacsn5* Widely Controls the Expression of CAZymes During Infection

**FIGURE 6:**
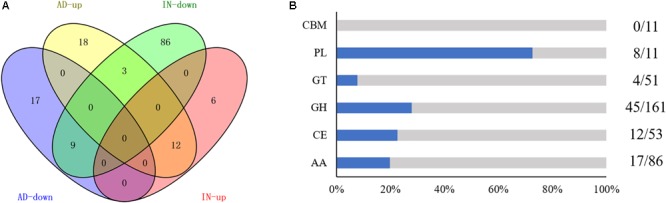
Differentially expressed CAZyme genes during AD and IN. **(A)** Venn diagram showing the upregulated and downregulated CAZyme genes in Δ*Aacsn5* mutant compared with the wildtype strain. **(B)** Percentage of exclusively down-regulated CAZyme genes (blue bars) in Δ*Aacsn5* mutant during infection. The corresponding number of exclusively downregulated CAZyme genes and the total number in the genome are shown on the right. GH, glycoside hydrolase; PL, polysaccharide lyase; CBM: carbohydrate-binding modules; GT: glycosyl transferase; CE: carbohydrate esterases; AA: auxiliary activities.

Carbohydrate-active enzymes (CAZymes) play important roles in the breakdown of complex carbohydrates and are responsible for the acquisition of nutrients from the plant for phytopathogenic fungi. Previously, a total of 373 putative CAZyme genes were identified in *A. alternata* Z7 (Wang et al., 2016). Markedly, 98 of them showed significantly decreased expression levels during infection after the deletion of *Aacsn5* (**Figure 6A** and Supplementary Table S6). Furthermore, 86 of these 98 CAZymes showed a unique expression pattern associated with pathogenicity, including 17 auxiliary activities, 12 carbohydrate esterases, 45 glycoside hydrolases, 4 glycosyl transferases (GTs), and 8 polysaccharide lyases (PLs) (**Figure 6B**). In particular, only 8% of the GT genes in the genome were downregulated in Δ*Aacsn5* during infection while 73% of the PL genes show a decreased expression level (**Figure 6B**). The transcript levels of 18 CAZymes were significantly elevated during infection while 33 and 26 CAZYmes overexpressed and underexpressed, respectively, during the asexual development (**Figure 6A** and Supplementary Table S6).

### *Aacsn5* Mediates the Expression of Some Transcription Factors

*Aacsn5* may play its regulatory role by interacting with transcription factors. We then investigated which transcription factors were differentially expressed in the Δ*Aacsn5* strain. We found that 11 transcription factor genes are upregulated and 9 are downregulated during asexual development in *Aacsn5* mutant while 12 and 6 transcription factors are upregulated and downregulated, respectively, during infection (Supplementary Table S7). Homologs of two functionally characterized C_2_H_2_-type transcription factors showed significantly downregulated gene expression in Δ*Aacsn5* during conidiation. One is the above-mentioned *flbC* (AALT_g5513, log2FC, -3.0), which is proposed to be involved in conidiophore development (Kwon et al., 2010), and the other is the *amdA* (AALT_g561, log2FC, -2.3), which controls the expression of the *amdS* and *aciA* structural genes required for acetamide catabolism (Lints et al., 1995). The nitrate-specific transcription factor NirA can activate the expression of the nitrate assimilation genes when nitrate or nitrite is present (Strauss et al., 1998). We discovered that the transcript level of its homolog (AALT_g8635, log2FC, 2.2) was increased in Δ*Aacsn5* mutant during asexual development. Interestingly, we found that the pH-responsive transcription factor PacC (AALT_g2998, log2FC, -2.1), whose homologs mediate not only the sensing and transduction of ambient pH but also the level of virulence in some plant pathogenic fungi (Caracuel et al., 2003; Zhang et al., 2013), was underexpressed in Δ*Aacsn5* during infection. The functions of the remaining transcription factors are largely uncharacterized and their relationship with *Aacsn5* needs further investigation.

### *Aacsn5* Regulates Many SM Gene Clusters During Conidiation and Infection

**FIGURE 7:**
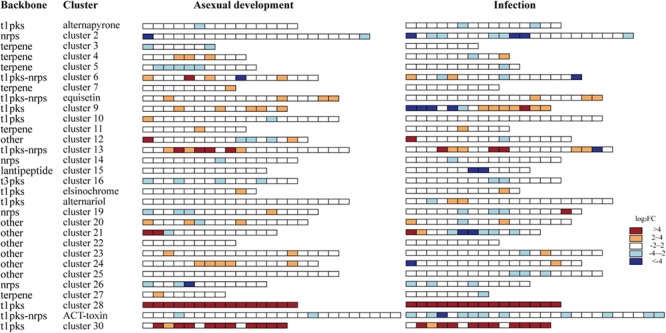
Differential expression of SM gene clusters in the Δ*Aacsn5* mutant during AD and IN. pks, polyketide synthase; nrps, non-ribosomal peptide synthetase; t1, Type 1; t3, Type 3.

To examine the relationship between SM and asexual development and pathogenicity mediated by *Aacsn5*, we examined the transcriptional responses of the 30 biosynthetic gene clusters in *A. alternata* predicted by anti-SMASH 4.0 (Blin et al., 2017; Supplementary Table S8). Overall, *Aacsn5* showed a broad regulation of SM gene clusters in both conditions (**Figure 7**). The transcript level of cluster 28 and 30 was strongly induced in both conditions (**Figure 7**), suggested a negative regulation of *Aacsn5* on both clusters. Clusters 15 and 25 are not regulated by *Aacsn5* during conidiation; however, expression of some genes in these clusters is evidently decreased during infection (**Figure 7**). Conversely, clusters 3 and 7 are not regulated by *Aacsn5* during infection while expression of a few genes in these clusters is underexpressed during conidiation (**Figure 7**). Only a few genes in clusters 2, 21, and ACT toxin were regulated by *Aacsn5* during asexual development; however, a large number of genes in these clusters showed significantly underexpressed mRNA level during infection (**Figure 7**). ACT toxin is known to be the determinant for the pathogenicity of *A. alternata* to citrus (Kohmoto et al., 1993; Wang et al., 2016), so the reduced biosynthesis of this critical factor can help to explain the phenotype of less virulent Δ*Aacsn5*. Only one or two genes in cluster 5 and 24 were regulated by *Aacsn5* during infection while a large number of genes in these clusters are significantly downregulated and upregulated, respectively, during conidiation (**Figure 7**). Finally, a mixed regulation mode (an SM cluster has both upregulated and downregulated genes) was found only within cluster 6 in Δ*Aacsn5* during conidiation while this was very common (found in 11 clusters) during infection (**Figure 7**). Our results indicate that *Aacsn5* regulates SM in a complex manner, which is associated with conidiation and infection.

### Roles of *Aacsn5* in Nutrients’ Utilization and Toxin Production During Development

**FIGURE 8:**
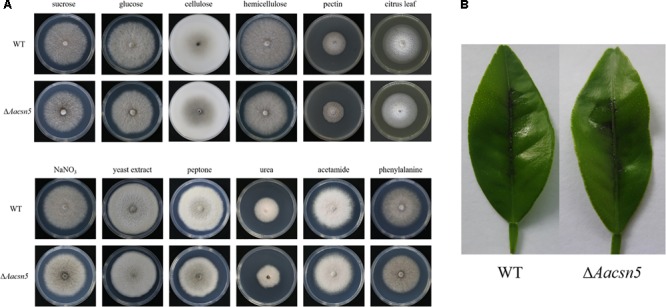
Roles of *Aacsn5* in nutrient utilization and toxin production during development. **(A)** Growth of the wildtype and Δ*Aacsn5* on different carbon or nitrogen sources. Mycelial plugs were taken from the wildtype and Δ*Aacsn5* and grown individually on Czapek’s medium with the glucose, sucrose, cellulose, hemicellulose, pectin, or citrus leaf as the sole carbon source or, with NaNO_3_, yeast extract, peptone, urea, acetamide, or phenylalanine as the sole nitrogen source at 25°C for 6 days. **(B)** Necrotic lesions appearing on detached citrus leaves inoculated by spraying with the crude ACT toxin extracts from both the wildtype and Δ*Aacsn5* in the axenic culture.

Our RNA-Seq data suggested that the carbohydrate and nitrogen metabolic processes in Δ*Aacsn5* were significantly affected during infection (Supplementary Table S3). To determine if those were caused by the defect of the Δ*Aacsn5* mutant in nutrition utilization during development, we performed growth arrays of the wildtype and the Δ*Aacsn5* mutant on different carbon and nitrogen sources. The results showed that the Δ*Aacsn5* exhibited a growth defect of 2–15% depending on different nutrients compared to the wildtype strain (**Figure 8A** and Supplementary Figure S5). Especially, when the citrus leaves were used as the sole carbon source, the growth inhibition rate of the Δ*Aacsn5* mutant was ∼8% (**Figure 8A** and Supplementary Figure S5).

The tangerine pathotype of *A. alternata* produces a host-selective ACT toxin which has been suggested as being the determining factor of pathogenicity (Miyamoto et al., 2008; Wang et al., 2016). Our transcriptome data also showed that the expression of the genes in the ACT gene cluster was significantly underexpressed during infection (**Figure 7** and Supplementary Table S9). qRT-PCR analysis confirmed that the ACTT2 (AALT_g11743) and the ACTTS3 gene (AALT_g11750), which were reported to be critical for ACT toxin production in the tangerine pathotype of *A. alternata* (Miyamoto et al., 2008; Miyamoto et al., 2010), were significantly downregulated in Δ*Aacsn5* during infection (Supplementary Figure S4). To test whether *Aacsn5* plays a role in regulating ACT production during development, we treated ponkan leaves with a dilution series of crude ACT toxin extracts from both wildtype and Δ*Aacsn5* strains in the axenic culture. The results showed that both strains induced similar necrotic lesions on the detached ponkan leaves (**Figure 8B** and Supplementary Figure S6).

Taken together, these results revealed that *Aacsn5* plays a limited or no role in nutrients utilization and ACT toxin production during development. Combined with the transcriptome analysis, our data suggested that there might exist a unique host’s immune response during the process of pathogen–plant interaction, causing the attenuated virulence of the Δ*Aacsn5* mutant.

## Discussion

As deficiency in any CSN subunits is lethal in higher eukaryotes, fungi are good model systems for an investigation into the molecular mechanisms of the CSN. Several studies have been performed to elucidate CSN composition, activity, and cellular functions in the model fungi including *S. cerevisiae, N. crassa*, and *A. nidulans* (Nahlik et al., 2010; Wang et al., 2010; Licursi et al., 2014). In this work, we investigated the biological roles that *csn5* plays in a plant pathogenic fungus, the tangerine pathotype of *A. alternata*, which is an economically important agent of the citrus brown spot disease worldwide.

The production of asexual spores is of vital importance in the life cycle of phytopathogenic fungi. One of the major findings we present here is that the formation of conidia is totally blocked in the tangerine pathotype of *A. alternata* after functional inactivation of the *csn5* homolog gene (**Figure 1B**), demonstrating that *Aacsn5* is absolutely required for conidiation. A similar phenotype was also observed in the *csn5* deletion mutant of another filamentous fungus, *Pestalotiopsis fici* (Zheng et al., 2017). However, conidiation was not affected in the *A. nidulans csn5* deletion strain, though this mutant showed a severe defect in the sexual developmental cycle (Busch et al., 2003; Nahlik et al., 2010). In *N. crassa*, the *csn5* mutant produces fewer conidia on race tubes than the wildtype (Wang et al., 2010). Taken together, these results indicate that the role of *csn5* in conidiation has markedly diverged in different fungi.

A transcriptome analysis was performed to unveil the regulatory role *Aacsn5* played during conidiation. Our results showed that genes downregulated in Δ*Aacsn5* during asexual development were significantly enriched in the transport-related processes and nitrogen metabolism (**Figure 4B**), and thus we speculate that the conidiation deficiency may be caused by failure to transport some nitrogen-containing compounds. Carbohydrate and nitrogen concentrations and their ratios can greatly influence the sporulation of many fungi (Elson et al., 1998; Gao et al., 2007). Further, SM is also an important factor for sporulation; the molecular mechanisms regulating SM are often involved in the control of asexual development (Keller, 2015; Macheleidt et al., 2016). For example, conidiogenone, an endogenous diterpenoid with conidiation-inducing activity, is continuously produced and released to the culture medium during the growth stage of *Penicillium cyclopium*, and it will trigger conidiation when it accumulates to a threshold concentration (Roncal et al., 2002). In our transcriptome data, we found that *Aacsn5* has a broad regulation of SM gene clusters; especially, the transcript level of cluster 28 and 30 was strongly induced in Δ*Aacsn5* (**Figure 7**). Although the products of most SM clusters are not known, we speculate that there might be a relationship between SM and *Aascn5*-mediated sporulation.

The Velvet complex is a global regulator of morphogenesis and SM in filamentous fungi (Bayram et al., 2008). Deletions of either of two members of this complex, *laeA* and *veA*, greatly reduce conidia production in *A. alternata* (Estiarte et al., 2016). However, the expression of these genes or of any other Velvet family proteins was not affected in the Δ*Aacsn5* mutant (Supplementary Table S5). We also examined the expression of genes involved in the sporulation in other fungi, which include the BrlA > AbaA > WetA central regulatory pathway, the light-dependent signaling pathway, the velvet family proteins, MAP kinase signaling pathway, G proteins, and FluG-mediated signaling pathway (Park and Yu, 2012), but almost all of the genes in these pathways showed no difference at the transcriptional level (Supplementary Table S5). These data suggested that the *Aacsn5* might mediate different mechanism underlying the sporulation from the important regulators in other fungi.

Our pathogenicity assays showed that the *A. alternata*Δ*Aacsn5* mutants induced no or significantly reduced necrotic lesions on detached citrus leaves (**Figure 3**), demonstrating that *Aacsn5* is required for wildtype levels of pathogenicity. To our knowledge, this is the first time that *csn5* has been reported to be involved in virulence in phytopathogenic fungi. Previously, both the production of host-specific ACT toxin and the ability to detoxify reactive oxygen species (ROS) have been shown to be critical factors for the pathogenicity of the *A. alternata* tangerine pathotype (Miyamoto et al., 2008; Lin et al., 2009). The ability to synthesize the ACT toxin in the Δ*Aacsn5* mutant was not affected during vegetative growth in liquid medium (**Figure 8B** and Supplementary Figure S6). A similar situation was also observed in the virulence-attenuated *A. alternata Yap1, Fus3, Hog1, Nps6*, and *Skn7* gene deletion mutants when grown *in vitro* (Lin et al., 2009, 2010; Lin and Chung, 2010; Chen et al., 2012, 2013). However, successfully synthesizing the ACT toxin *in vitro* does not guarantee an equivalent ACT toxin production in those mutants during infection. In those studies, no evidence was provided about whether the biosynthesis of ACT toxin *in planta* was impaired. Our transcriptome data showed that the expression of the genes in the ACT gene cluster was significantly underexpressed during infection (**Figure 7**), strongly suggesting the repression of the ACT toxin biosynthesis *in planta*. The discrepancy of toxin production between *in vitro* and *in planta* could be attributed to the host’s immune response during the process of pathogen–host interaction, causing the difficulty of Δ*Aacsn5* mutant in expressing those genes responsible for the ACT toxin biosynthesis, which coincides with the virulence assay results. Additionally, the expression of other clusters like cluster 2, 9, 15, 21, and 25 showed a very different expression pattern during infection compared to expression during asexual development (**Figure 7**), suggesting that these SM clusters might also be related to the pathogenicity of *A. alternata*. Our growth assays showed no change in the tolerance to H_2_O_2_ and menadione in the Δ*Aacsn5* strain compared to wildtype (**Figure 2**), suggesting that *Aacsn5* plays no role in resistance to oxidative stress tolerance. Previously, two transcription factors SKN7 (AALT_g8622; Chen et al., 2012) and YAP1 (AALT_g912; Lin et al., 2009), HOG1 kinase (AALT_g10096; Lin and Chung, 2010), three NADPH oxidases NoxA (AALT_g5618; Yang and Chung, 2012), NoxB (AALT_g6028), and NoxR (AALT_g5214; Yang and Chung, 2013), glutathione peroxidase Gpx3 (AALT_g41; Yang et al., 2016), and major facilitator superfamily transporter Mfs19 (AALT_g2684; Chen et al., 2017) were shown to be essential for the detoxification of cellular stresses induced by ROS and for the pathogenesis in citrus. However, none of these genes showed significantly different expression in the *Aacsn5* deletion mutants during infection (Supplementary Table S3). Thus, the reduced virulence of Δ*Aacsn5* is not associated with ROS scavenging.

In necrotrophic fungi, breaking down dead tissues for nutrient acquisition is carried out by various CAZymes and is one important process associated with pathogenesis. In our transcriptome data, we identified that a large number (86/373) of CAZymes encoding genes showed uniquely decreased expression in Δ*Aacsn5* mutant during infection (**Figure 6**), indicating that *Aacsn5* positively regulates CAZymes during the infection of *A. alternata* to citrus leaves. Nitrogen availability and type can also be important factors for the virulence of fungal pathogens (Bolton and Thomma, 2008). For example, the *A. alternata* tobacco pathotype utilizes ammonia as a stimulator to form infection structures and to switch to a necrotrophic lifestyle (Duan et al., 2010). Studies using nitrogen-starved conditions to mimic the environment that a pathogen encounters during growth in planta have discovered a subset of genes related to fungal virulence (Stephenson et al., 2000; Donofrio et al., 2006; Bolton and Thomma, 2008; Brown et al., 2008). In this research, several amino acid metabolism pathways were identified to be underexpressed in Δ*Aacsn5* mutant during infection (Supplementary Table S3), implying the role of *Aacsn5* in regulating nitrogen metabolism during infection.

Taken together, our results demonstrate that Aacsn5 plays a critical role in the conidiation and pathogenicity in the pathotype of *A. alternata*, the causal agent of Alternaria brown spot disease in citrus worldwide. To explore the potential mechanisms of *Aacsn5* in regulating conidiation and pathogenicity, we performed RNA-Seq analyses and discovered candidate genes associated with conidiation and pathogenesis. Our analysis opens avenues into researches on the molecular function of the CSN in plant pathogenetic fungi. Ongoing research is needed to elucidate the precise molecular mechanism by which the CSN affects fungal development, nutrition metabolism, SM, and pathogenicity.

## Author Contributions

MW and HL conceived and designed the experiments. XY, MW, RR, and HF performed the experiments. MW analyzed the data and wrote the paper. All authors reviewed the manuscript.

## Conflict of Interest Statement

The authors declare that the research was conducted in the absence of any commercial or financial relationships that could be construed as a potential conflict of interest.
