# Supplementary material for: Csn5 Is Required for the Conidiogenesis and Pathogenesis of the Alternaria alternata Tangerine Pathotype
Source: Front Microbiol. 2018 Mar 20;9:508. doi: 10.3389/fmicb.2018.00508 (PMC5870056; doi:10.3389/fmicb.2018.00508)
Supplement: Supplementary file 1 [file Presentation_1.PDF]

Supplementary Figure 1

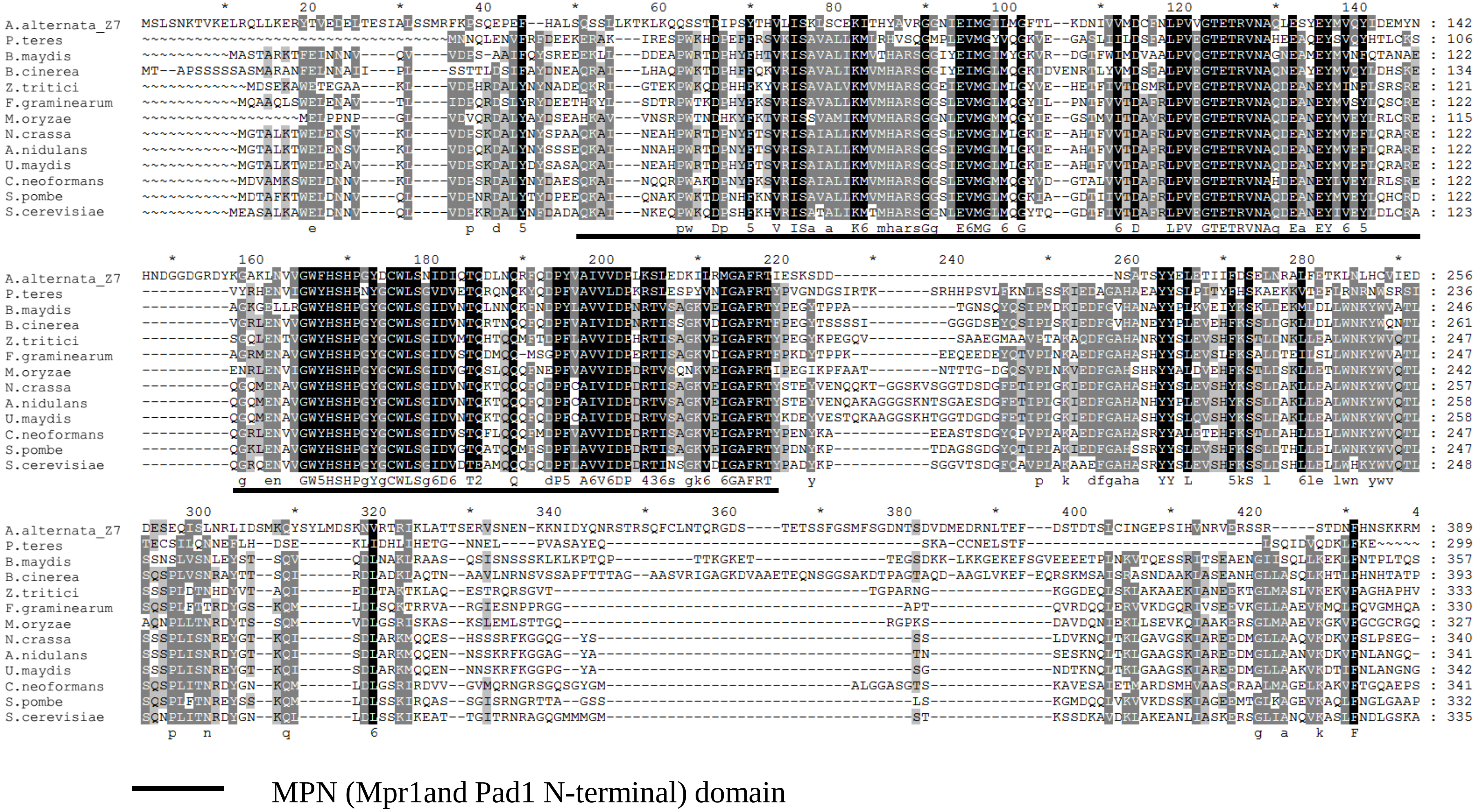

Fig. S1 Alignment of CSN5 orthologs in different fungal species.

Supplementary Figure 2

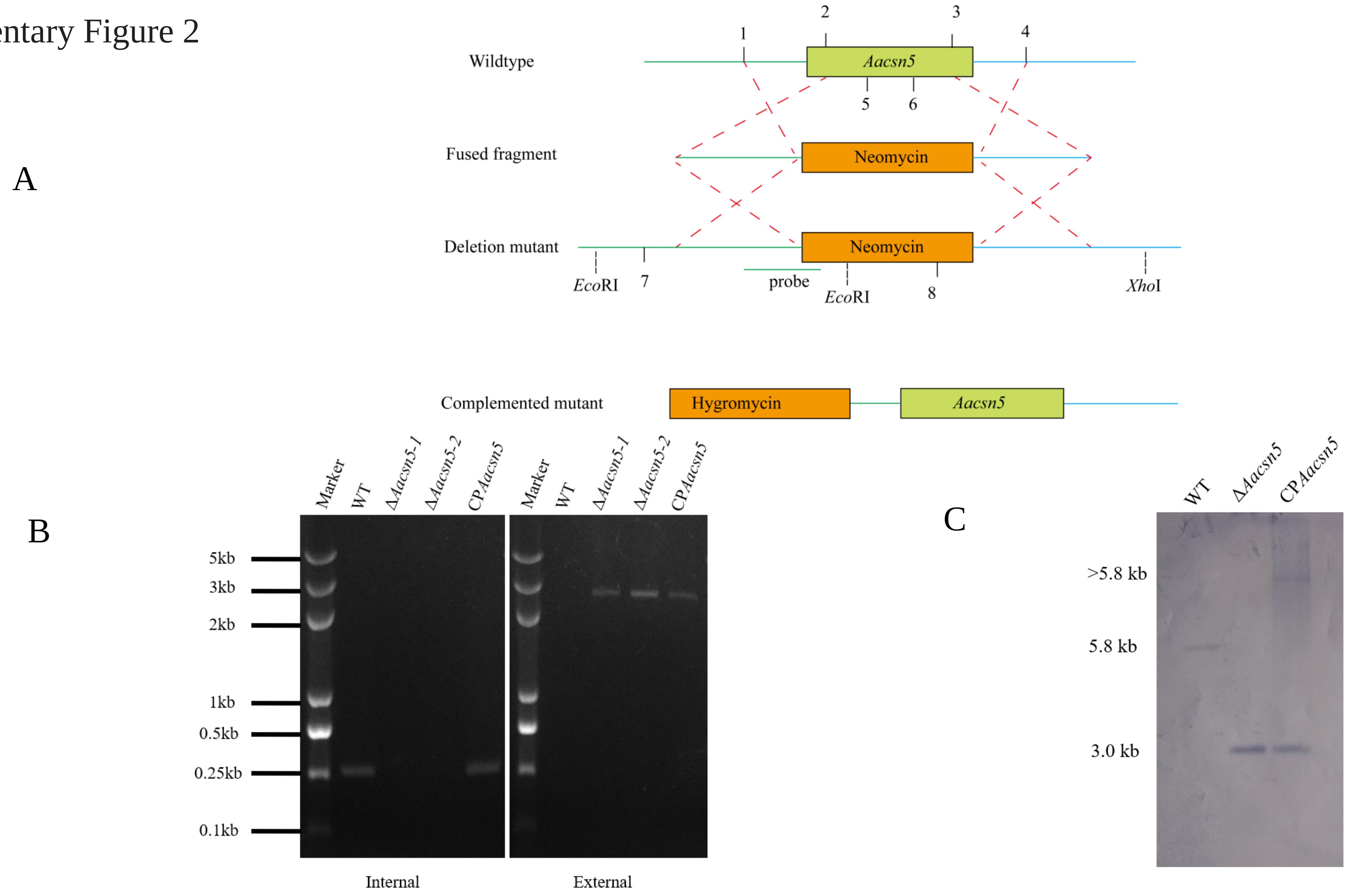

Fig. S2 Construction and confirmation of *Aacsn5* disrupted and complemented mutants. (A) Gene replacement strategy for *Aacsn5*. Numbers indicate primers listed in Table S1. Verification of the positive transformants by polymerase chain reaction (PCR) (B) and southern blot analysis (C). Genomic DNAs of the wildtype and the mutant strains were digested with *Eco*RI and *Xho*I. Both transformants had a 3.0 kb band, but lacked a 5.8 kb one present in the wildtype, the complemented mutant had an additional band much larger than 5.8 kb.

Supplementary Figure 3

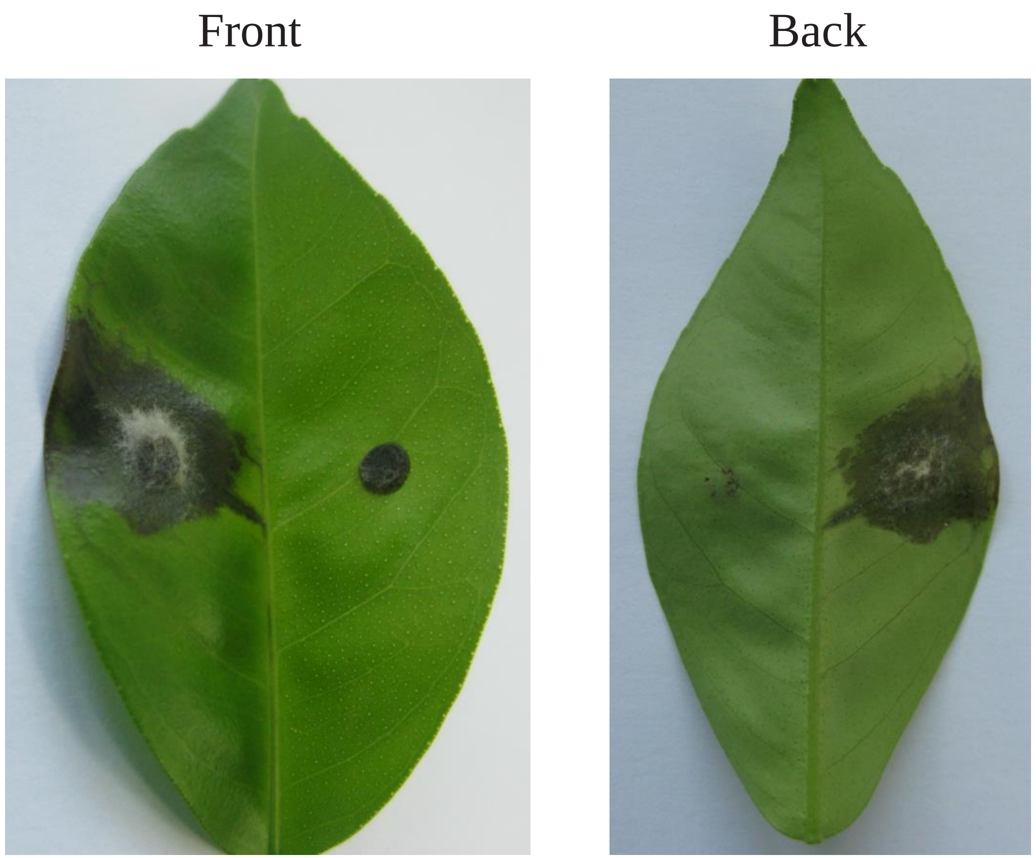

WT | Δ*Aacsn5* Δ*Aacsn5* | WT

Fig. S3 Pathogenicity assays on detached leaves with wounding prior to inoculation.

Supplementary Figure 4

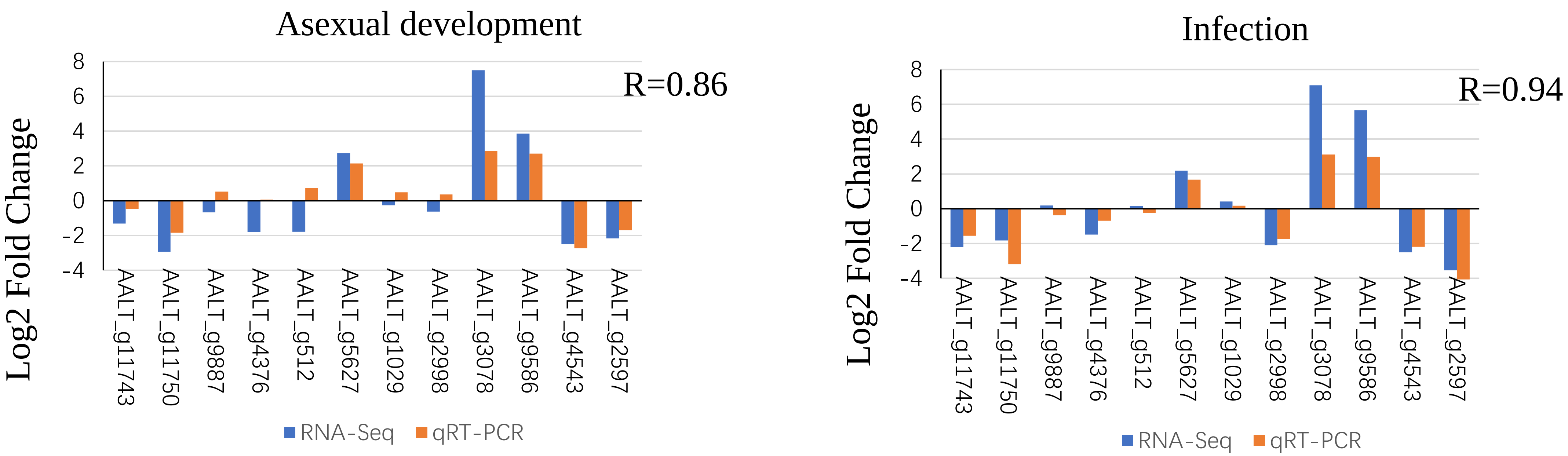

Fig. S4 Validation of the RNA-seq data using qRT-PCR. Fold change expression levels were normalized to actin expression levels in each sample and data is presented relative to wildtype expression levels using $2^{-\Delta\Delta Ct}$ method. Pearson coefficient correlation analysis was performed to compare fold changes of transcripts measured by RNA-seq and qRT-PCR.

Supplementary Figure 5

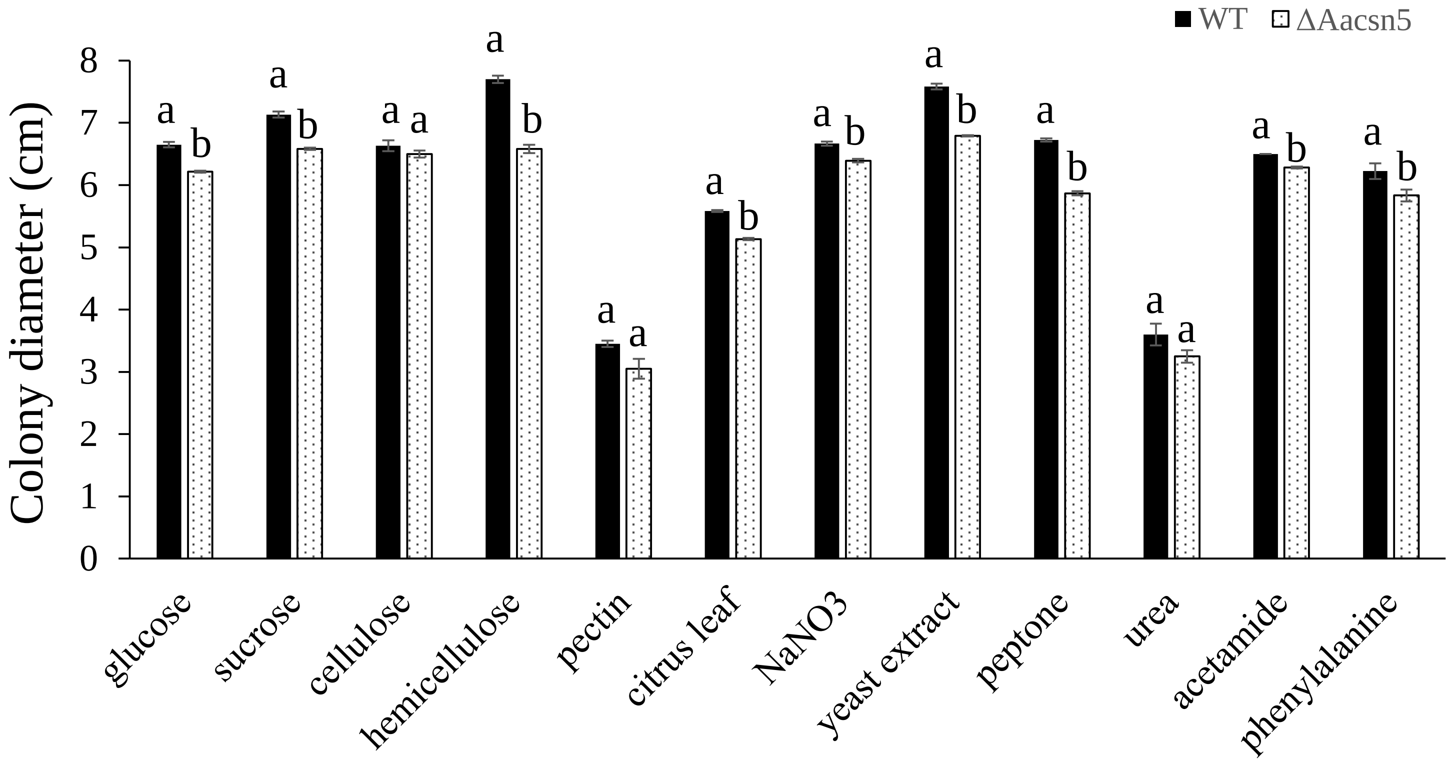

Fig. S5 Colony diameter of the wildtype and Δ*Aacsn5* on different carbon or nitrogen sources indicated in the figure. All plates were incubated for 6 days at 25 °C. Different letters are used to mark significantly differences from one another as determined by Student's t test ($P < 0.05$) in each treatment.

Supplementary Figure 6

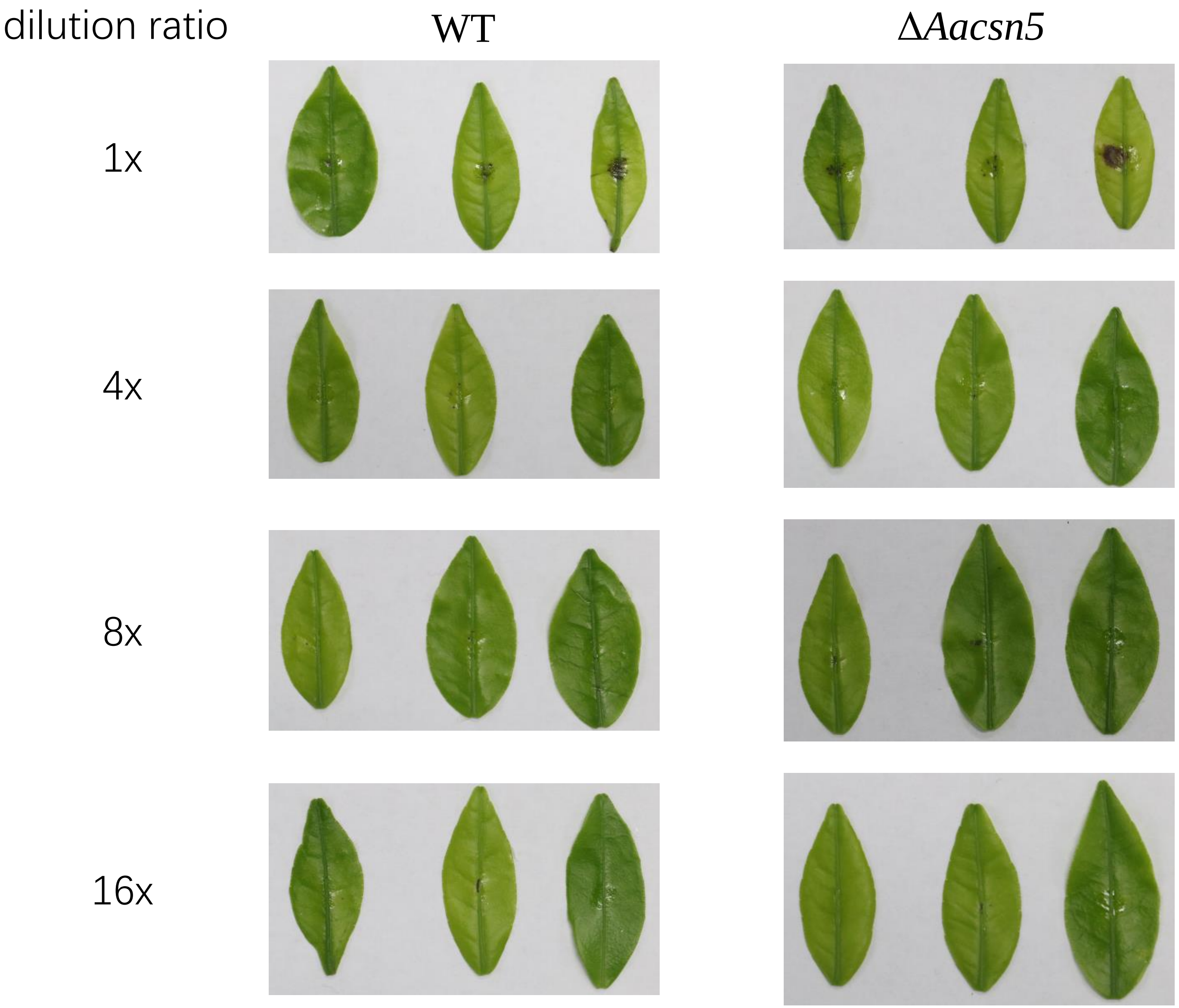

Fig. S6 Necrotic lesions appearing on detached citrus leaves inoculated by spraying with a dilution series of the crude ACT toxin extracts from both the wildtype and Δ*Aacsn5* in the axenic culture.
